# Prevalence and pattern of dyslipidemia in Nepalese individuals with type 2 diabetes

**DOI:** 10.1186/s13104-017-2465-4

**Published:** 2017-04-04

**Authors:** Daya Ram Pokharel, Dipendra Khadka, Manoj Sigdel, Naval Kishor Yadav, Shreedhar Acharya, Ramchandra Kafle, Ravindra Mohan Sapkota, Tara Sigdel

**Affiliations:** 1 Department of Biochemistry, Manipal College of Medical Sciences, Deep Height, Pokhara-16, Kaski, Nepal; 2 Department of Laboratory, School of Health and Allied Sciences, Pokhara University, Dhungepatan, Lekhnath, Kaski Nepal; 3 Assessment and Evaluation Division, Northern Ontario School of Medicine, 935 Ramsey Lake Road, Sudbury, ON P3E 2C6 Canada; 4 Department of Internal Medicine, Manipal College of Medical Sciences and Teaching Hospital, Phulbari, Pokhara, Kaski Nepal; 5 Shikhar Biotech Pvt Ltd, Khumaltar, Lalitpur Nepal; 6 Division of Transplant Surgery, Department of Surgery, University of California San Francisco, San Francisco, CA 94017 USA

**Keywords:** Type 2 diabetes, Dyslipidemia, Cardiovascular disease, Prevalence, Pokhara, Nepal

## Abstract

**Background:**

Atherogenic dyslipidemia is an important modifiable risk factor for cardiovascular disease among patients of type 2 diabetes mellitus. Timely detection and characterization of this condition help clinicians estimate future risk of cardiovascular disease and take appropriate preventive measures. The aim of this study was to determine the prevalence, pattern and predictors of dyslipidemia in a cohort of Nepalese patients with type 2 diabetes.

**Results:**

We found mixed dyslipidemia as the most prevalent (88.1%) and isolated dyslipidemia (10.1%) as the least prevalent forms of dyslipidemia in our patients. The most prevalent form of single dyslipidemia was high LDL-C (73.8%) and combined dyslipidemia was high TG, high LDL-C and low HDL-C (44.7%). Prevalence of all single and mixed dyslipidemia was higher in patients with poor glycemic control and hypertension. The glycemic status of patients correlated with their fasting serum lipid profile. Dyslipidemia was associated mainly with male gender, poor glycemic control and hypertension.

**Conclusions:**

Atherogenic dyslipidemia is associated mainly with male gender, poor glycemic control and hypertension. It is highly prevalent in Nepalese patients with type 2 diabetes. Urgent lifestyle modification, sustained glycemic control and aggressive lipid lowering treatment plans are necessary to minimize the future risk of cardiovascular disease in this population.

**Electronic supplementary material:**

The online version of this article (doi:10.1186/s13104-017-2465-4) contains supplementary material, which is available to authorized users.

## Background

Type 2 diabetes mellitus (T2DM) is the third major non-communicable disease in Nepal, and is approaching pandemic levels due to rapid change in socioeconomic status and life-style of the people [1]. T2DM amplifies the risk of cardiovascular disease (CVD) several fold, making it a significant risk factor of the latter. More than 50% patients with T2DM die due to coronary heart disease (CHD) [2, 3]. Among several modifiable and non-modifiable risk factors for CVD, T2DM is the strongest, as it is strongly associated with atherogenic dyslipidemia [4, 5]. The atherogenic dyslipidemia in diabetic patients results from insulin deficiency or resistance that promotes lipolysis in the visceral adipocytes and increases the flux of free fatty acids in plasma and liver. Moreover, the activity of an endothelial enzyme, lipoprotein lipase, also decreases. These conditions lead to hepatic steatosis, over-secretion of larger triglyceride (TG)-rich very low density lipoprotein 1(VLDL1) particles into the plasma, over-secretion of hepatic apolipoprotein B (ApoB), impaired clearance of chylomicrons and decreased receptor mediated endocytosis in the liver [6, 7]. The most common phenotypic pattern of diabetic dyslipidemia involves lipid triad with raised triglycerides, reduced high density lipoprotein cholesterol (HDL-C) and increased concentration of small, dense low density lipoprotein cholesterol (LDL-C) particles [8, 9]. Additionally, total cholesterol (TC)/HDL-C ratio, non-HDL-C and ApoB have also been shown to be directly involved in the atherogenic process and development of CVD [2, 10]. Diabetic dyslipidemia has therefore emerged as an important biomarker for the increased CVD risk observed in diabetic patients. Significant reduction of CVD related morbidity and mortality by lipid-lowering agents such as statins underscores their importance in the cardiovascular health of diabetic patients [11]. Therefore, early detection and aggressive management of dyslipidemia are very important in saving the lives of diabetic patients from atherogenic cardiovascular diseases.

The Western hilly region of Nepal is mostly populated by ethnic groups like Gurung, Magars and Dalits which differentiates this population genetically from other areas of Nepal. The socioeconomic status, life-style, dietary habit and cultural practices of these ethnic groups make them more vulnerable to high incidence of CVD than any other ethnic groups in this region. Few recent hospital based studies and our own clinical observations have shown that the prevalence of metabolic syndrome and cardiovascular disease is very high in this region particularly among these three ethnic groups [12, 13]. Atherogenic dyslipidemia is a major modifiable risk factor of CVD. However, no systematic study has been carried out so far in this region to map the actual epidemiology of CVD risk factors including the dyslipidemia. Given that the prevalence and pattern of CVD risk factors differ according to geographic location, ethnicity, dietary habits and socio-economic status of the population under study, we hypothesize that epidemiology of dyslipidemia is different for the population of this region compared to others. Our study thus aims to describe the prevalence, pattern and independent predictors of dyslipidemia among type 2 diabetic patients of Western hilly region of Nepal.

## Methods

### Study design

We conducted a cross-sectional study on patients with type 2 diabetes aged 30–74 years from July 2013 to December 2014.

### Study setting

The study was carried out at the Manipal Teaching Hospital (MTH), Pokhara Nepal which is one of the largest multi-specialty tertiary care hospital attached to Manipal College of Medical Sciences (MCOMS). This hospital provides clinical trainings to both Nursing and Medical students in addition to providing comprehensive healthcare services to the general public of hilly districts of Western Development Region of Nepal.

### Sample size and selection criteria

We enrolled a total of 497 diabetic patients originating mainly from Gandaki, Dhaulagiri, Lumbini zones of the Western Development Region of Nepal. They were randomly selected from the list of outpatients who were clinically examined in medicine and other outpatient departments and approached the sample collection unit of MTH to have their blood glucose, HbA1c, lipid profile and other parameters measured. All those randomly selected diabetic patients who provided informed consent were enrolled in the study without regard to their treatment for dyslipidemia. The presence of diabetes was confirmed based on their previous medical records, clinical examination and past or current laboratory results. Repeated inclusion of the same patients was avoided by using a filter that consisted of their unique hospital number, full name and age.

### Interview, anthropometry and measurement of physiological variables

The demographic data of the patients including their personal and family medical history, smoking and dietary habit were collected by interviewing them with a pre-validated set of questionnaire (see Additional file 1). Anthropometric parameters such as height, weight, body mass index (BMI), waist circumference and blood pressures were measured following standard protocols.

### Measurements of biochemical variables

Following the interview and anthropometry, patients were asked to return the next morning for blood collection in overnight (≥8 h) fasting state. Five ml venous blood was drawn from each subject and then divided into fluoride-oxalate, ethylene diamine tetraacetate (EDTA) and plain test tubes. All direct biochemical measurements were made using automated chemistry analyzer and ready-to-use reagent kits according to the standardized protocols provided by the manufacturers (Erbachem XL-300, Germany). All tests were run in duplicate and appropriate standards and quality control sera were used to ensure the accuracy of the measurements. Fasting glucose was measured in fluoridated plasma by glucose oxidase/peroxidase method. TC and TG were estimated by cholesterol oxidase/peroxidase and glycerol phosphate kinase methods, respectively. HDL-C was measured by phosphotungstate precipitation method. The value of LDL-C was calculated using Friedwald formula [14]. Non-HDL-C was calculated by subtracting HDL-C from TC. Total ApoB was estimated using the following equations: ApoB = 0.65 × TC−0.59 × HDL-C + 0.01 × TG when TG < 270 mg/dl and ApoB = 25.6 + 0.58 × TC−0.38 × HDL-C−0.06 × TG when TG > 270 mg/dl [15]. Glycated hemoglobin (HbA1c) was measured on EDTA blood by ion-exchange resin method.

### Definitions

Dyslipidemia was defined according to the third report of National Cholesterol Education Program Adult Treatment Panel (NCEP ATP III) criteria [2] with the following cut off values: hypercholesterolemia-serum TC level ≥200 mg/dl; hypertriglyceridemia–serum TG level ≥150 mg/dl; low HDL-C–HDL-C level ≤40 mg/dl for both men and women; high LDL-C–LDL-C level ≥100 mg/dl and high TC/HDL-C ratio ≥5, high non-HDL-C ≥ 130 mg/dl and high ApoB ≥ 90 mg/dl. Mixed or combined dyslipidemia was defined as the combination of two or more single dyslipidemia mentioned before. Isolated dyslipidemia was defined as follows: isolated hypercholesterolemia-serum TC ≥ 200 mg/dl and TG < 150 mg/dl; isolated hypertriglyceridemia–serum TG ≥ 150 mg/dl and TC < 200 mg/dl; isolated low HDL-C–HDL-C < 40 mg/dl (male and female) without hypertriglyceridemia, hypercholesterolemia and high LDL-C; and isolated high LDL-C–LDL-C > 100 mg/dl without hypertriglyceridemia, hypercholesterolemia or low HDL-C.

Patients were designated as to having type 2 diabetes when they had already diagnosed by a physician at the age of 30 or above and on oral hypoglycemic drugs and/or those who had plasma glucose levels above the cut off values recommended by the WHO guidelines i.e. fasting blood glucose ≥126 mg/dl and/or 2 h post meal or random blood glucose value ≥200 mg/dl [16]. Hypertension was deemed to be present when patients were already diagnosed by a physician and were on anti-hypertensive medications and/or those who had systolic blood pressure ≥140 mmHg and/or diastolic blood pressure ≥90 mmHg as suggested by the Joint National Committee 7 (JNC7) criteria [17]. Generalized and central obesity were defined according to the Asia Pacific guidelines for South Asians [18]. According to this, patients were categorized as overweight if their BMI was between 23 and 25 kg/m^2^ and obese if their BMI was ≥25 kg/m^2^. Central obesity was defined as waist circumference ≥90 cm (males), ≥80 cm (females). Poor diabetic control was defined as HBA1c > 7%.

### Statistical analyses

The data were analyzed using Microsoft Excel 2007 and SPSS for Windows version 17.0 (SPSS, Inc., Chicago, IL). Continuous variables were reported as mean ± standard deviation (SD) and categorical variables were reported in numbers and/or percentage. The Student’s t test was used to compare two continuous variables, and Chi square test was used to compare categorical variables. One-way analysis of variance (ANOVA) was used to compare three or more subgroups of a continuous variable. Pearson’s bivariate correlation analysis was performed to assess the correlation between dyslipidemia and other independent variables. Multivariate logistic regression was carried out to identify the risk factors that are independently associated with single and mixed dyslipidemia. Statistical significance was set at a p value <0.05.

## Results

A total of 497 type 2 diabetic patients, 36.2% females and 63.8% males, were enrolled in the present study, Their mean age was 52.7 ± 10.5 years while mean diabetes duration was 5.1 ± 3.8 years. Among these, 176 (35.4%) patients were not taking any anti-diabetic drugs, 282 (56.7%) were taking only oral hypoglycemic drugs, 26 (5.2%) were taking both oral hypoglycemic drugs and insulin, and remaining 13 (2.6%) were taking only insulin for controlling their blood glucose levels. Their demographic, anthropometric and biochemical characteristics are presented in Table 1. Males were significantly (p < 0.010) older, overweight or obese than females. Majority of the patients were urban residents (74.2%), non-smokers (72.8%) and non-vegetarians (92.8%). Prevalence of smoking habit (36.9%), general obesity (36.3%) and central obesity (51.7%) was significantly higher in males than in females (p < 0.010). There were 24.1% patients with poor glycemic control (HbA1c > 7.0%). The fasting plasma glucose level, duration of diabetes and hypertension, glycemic status and blood pressures did not differ significantly between males and females (p > 0.050).

**Table 1:** General and biochemical characteristics of the diabetic patients

Characteristics	Female (n = 180)	Male (n = 317)	p value	Total (n = 497)
Age (year)	55.6 ± 9.2	51.2 ± 10.9	0.000	52.7 ± 10.5
BMI (kg/m^2^)	23.9 ± 2.7	24.3 ± 2.2	0.069	24.2 ± 2.4
Overweight	68 (37.8)	126 (39.7)	0.003	194 (39.0)
Obese	45 (25.0)	115 (36.3)		160 (32.2)
Waist circumference (cm)	90.2 ± 8.0	94.5 ± 6.7	0.000	92.9 ± 7.5
Centrally obese	170 (94.4)	257 (81.1)	0.000	427 (85.9)
Residence				
Village	47 (26.1)	81 (25.6)	0.886	128 (25.8)
Urban	133 (73.9)	236 (74.4)		369 (74.2)
Current smokers	18 (10.0)	117 (36.9)	0.000	135 (27.2)
Diet				
Vegetarian	19 (10.6)	17 (5.4)	0.032	36 (7.2)
Non-vegetarian	161 (89.4)	300 (94.6)		461 (92.8)
Fating plasma glucose (mg/dl)	138.2 ± 40.6	134.2 ± 44.0	0.314	135.7 ± 42.8
DM duration (year)	5.3 ± 3.8	4.9 ± 3.9	0.224	5.1 ± 3.8
HbA1c (%)	6.4 ± 0.9	6.4 ± 0.9	0.932	6.4 ± 0.9
Glycemic control				
Good (HbA1c<7%)	138 (76.7)	239 (75.4)	0.750	377 (75.9)
Poor (HbA1C>7%)	42 (23.3)	78 (24.6)		120 (24.1)
SBP (mmHg)	125.1 ± 14.0	125.8 ± 11.6	0.562	125.5 ± 12.5
DBP (mmHg)	80.9 ± 9.2	82.2 ± 7.6	0.107	81.7 ± 8.2
Hypertension	75 (41.7)	130 (41.0)	0.003	205 (41.2)
Duration of HTN (year)	1.9 ± 3.6	1.6 ± 3.6	0.345	1.7 ± 3.6

The results are presented as mean ± SD for continuous variables and n (%) for categorical variables

^a^ p < 0.001

^b^ p < 0.05

^c^ p > 0.05 (two tailed); groups were compared using Students t test for quantitative variables and Chi square test for categorical variables

**Fig. 1:**
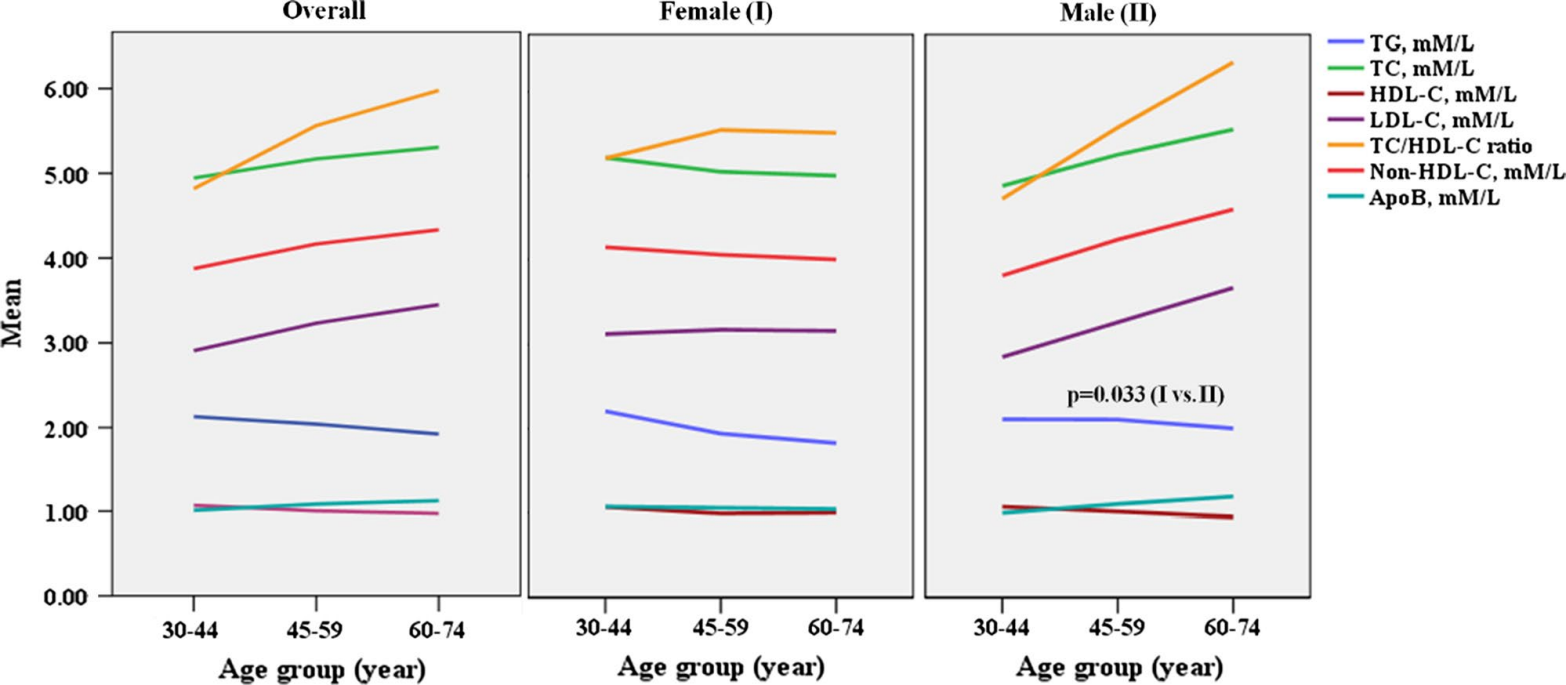
Age- and sex specific mean values of serum lipid parameters and their ratio in diabetic patients. Student's t-test was used to compare the group means of female (I) and male (II) patients. One-way ANOVA was used for comparing serum levels of single lipid parameter within three different age groups (30–44, 45–59 and 60–74). ***p for trend <0.001, **p for trend <0.010 *p for trend <0.05 (two tailed). TG, triglycerides; TC, total cholesterol; HDL-C, high density lipoprotein cholesterol; LDL-C, low density lipoprotein cholesterol; ApoB, apolipoprotein B. Mean refers to the serum mean concentrations and ratio of lipid parameters

The age- and sex specific values of serum lipid parameters are presented in Fig. 1. Among all the measured lipid parameters, only the serum TG level was higher in males (p < 0.050). The serum TG and HDL-C levels decreased while other lipid parameters increased with age. Gender-wise analysis showed that such age-specific variation of serum lipid parameters was more obvious in males than in females. Serum lipid levels and ratio were either unchanged or decreased with age in female patients. A one-way between-groups analysis of variance was conducted to explore the impact of age on serum lipid parameters. The difference in concentrations and ratio of serum lipid parameters for the three age groups (30–44, 45–59 and 60–74 years) was statistically significant except for TG. Post-hoc comparisons using the Tukey test indicated that the mean serum levels of TC, HDL-C, LDL-C, non-HDL-C and ApoB and TC/HDL-C ratio for age group 30–44 years were significantly different (p < 0.050) from age groups 45–59 and 60–64 years. Serum LDL-C concentration and TC/HDL-C ratio of age group 45–59 years differed from either age groups while serum concentrations of TC and non-HDL-C differed only with age group 30–44 years. No age group specific variation was found in the serum lipid parameters for females. In males, the serum concentrations of TC, LDL-C, non-HDL-C, ApoB and TC/HDL-C ratio of age group 30–44 years differed significantly from age groups 45–59 and 60–74 years. Only LDL-C concentration of age group 45–59 years differed significantly from age groups 30–44 and 60–74 years. Serum TC, non-HDL-C, ApoB concentrations and TC/HDL-C ratio of age group 45–59 years differed significantly only from age group 30–44 years.

Prevalence of single and mixed dyslipidemia has been presented in Table 2. The most prevalent single lipid disorder was increased non-HDL-C (75.5%) while the least prevalent was hypercholesterolemia (43.7%). Prevalence of mixed dyslipidemia was 88.1%. Prevalence of high LDL-C, non-HDL-C, ApoB and mixed dyslipidemia was significantly higher among males (p < 0.05).

**Table 2:** Age-and sex specific prevalence of single and mixed dyslipidemia in diabetic patients

	Overall				Female				Male				p value
	Total	30–44	45–59	60–74	Total	30–44	45–59	60–74	Total	30–44	45–59	60–74	
n	497	123	214	160	180	24	85	71	317	99	129	89	
Hypertriglyceridemia (%)	63.8	67.5	68.2	55.0**	60.0	70.8	63.5	52.1	65.9	66.7	71.3	57.3	0.186
Hypercholesterolemia (%)	43.7	35	44.9	48.8	41.1	54.2	37.6	40.8	45.1	30.3	49.6	55.1***	0.388
Low HDL-C (%)	44.5	35	48.1	46.9	45.0	41.7	48.2	42.3	44.2	33.3	48.1	40.6	0.744
High LDL-C (%)	73.8	69.9	75.2	75.0	68.3	70.8	69.4	66.2	77.0	69.7	79.1	82.0	0.035
High TC/HDL-C ratio (%)	45.3	35.8	48.1	48.8	43.9	41.7	44.7	43.7	46.1	34.3	50.4	52.8*	0.641
High non-HDL-C (%)	75.5	74.0	78.0	73.1	70.0	79.2	72.9	63.4	78.5	72.7	81.4	80.9	0.033
High ApoB (%)	73.6	72.4	76.6	70.6	67.8	79.2	68.2	63.4	77.0	70.7	82.2	76.4	0.025
Mixed dyslipidemia (%)	88.1	87.0	91.1	85.0	88.2	83.3	88.2	78.9	93.0	87.9	93.0	89.9	0.028

p for trend: ^***^ p < 0.001, ^**^ p < 0.010 ^*^ p < 0.05 (two tailed) compared to other subgroups within a category using ANOVA; the group means between male and females were compared using Student’s t-test. Dyslipidemia was diagnosed using NCEP ATP III guidelines- hypercholesterolemia: total cholesterol (TC) ≥200 mg/dl; hypertriglyceridemia: triglycerides (TG) ≥150 mg/dl; low HDL cholesterol (HDL-C): HDL-C < 40 mg/dl (both males and females); high LDL cholesterol (LDL-C): LDL-C ≥ 100 mg/dl; high total cholesterol: HDL-C ratio ≥5; High non-HDL-C: non-HDL-C > 130 mg/dl; High apolipoprotein B (ApoB): ApoB > 90 mg/dl; mixed dyslipidemia: when one or more of these lipid parameters were above the cut off values mentioned above

**Fig. 2:**
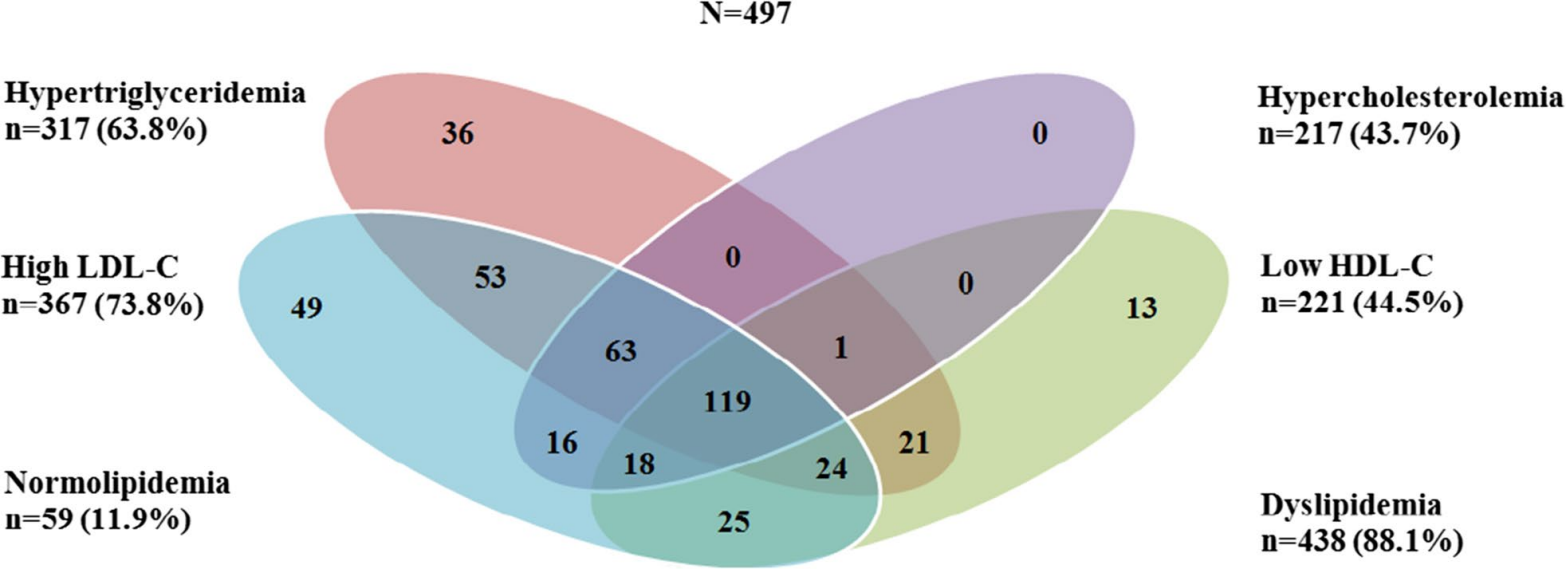
Venn diagram showing the overlapping of the individual components of dyslipidemia (hypertriglyceridemia, hypercholesterolemia, high LDL-C and low HDL-C) in diabetic patients

The pattern of dyslipidemia is shown in Fig. 2. Among dyslipidemic patients, there were 36 (8.2%) cases of isolated hypertriglyceridemia, 49 (11.2%) cases of isolated high LDL-C and 13 (3.0%) cases of isolated low HDL-C and no cases of isolated hypercholesterolemia. Likewise, there were 53 (12.1%) cases of combined hypertriglyceridemia and high LDL-C, 21 (4.8%) cases of combined hypertriglyceridemia and low HDL-C, 25 (5.7%) cases of combined high LDL-C and low HDL-C, 24 (5.5%) cases of combined hypertriglyceridemia, low HDL-C and high LDL-C and 119 (27.2%) cases of combined hypertriglyceridemia, hypercholesterolemia, low HDL-C and high LDL-C. Additionally, there were 63 (14.4%) cases of hypercholesterolemia, hypertriglyceridemia and high LDL-C, 16 (3.7%) cases of hypercholesterolemia and high LDL-C, 18 (4.1) case of hypercholesterolemia, low HDL-C and high LDL-C, and 1(0.2%) case of hypercholesterolemia, hypertriglyceridemia and low HDL-C. Prevalence of all single and mixed dyslipidemia was significantly higher (p < 0.001 for all) in patients with poor glycemic control and hypertension. Table 3 presents the prevalence of single and mixed dyslipidemia in diabetic patients based on their characteristics such as duration of diabetes, place of residence, smoking habit, glycemic status and blood pressure. Prevalence of low HDL-C and high TC/HDL-C ratio was significantly higher (p < 0.050) in patients with longer duration of diabetes. Only the prevalence of hypertriglyceridemia was significantly higher (p < 0.050) in non-smoker patients.

**Table 3:** Prevalence of single and mixed dyslipidemia in diabetic patients based on various characteristics of diabetic patients

	n	High TG (%)	High TC (%)	Low HDL-C (%)	High LDL-C (%)	High TC/HDL-C (%)	High non-HDL-C (%)	High ApoB	Mixed dyslipidemia
BMI (kg/m^2^)									
<25	337	58.8	40.9	43.0	70.9	42.4	73.3	71.8	83.1
≥25	160	74.4	49.4	47.5	80.0	51.3	80.0	77.5	92.5
p value		0.001	0.077	0.212	0.031	0.065	0.105	0.179	0.005
Duration of DM (years)									
≤7	360	64.4	40.8	39.7	73.6	40.8	77.2	75.3	86.4
7–13	123	61.0	49.6	56.9	74.0	56.9	69.9	69.1	85.4
≥14	14	71.4	64.3	57.1	78.6	57.1	78.6	71.4	85.7
p value		0.656	0.069	0.016	0.917	0.006	0.257	0.399	0.960
Place of residence									
Village	128	65.6	46.9	42.2	77.3	43.8	79.7	78.1	87.5
Urban	369	63.1	42.5	45.3	72.6	45.8	74.0	72.1	85.6
p value		0.615	0.395	0.691	0.296	0.688	0.196	0.182	0.599
Current smoker									
No	135	71.9	41.5	45.2	73.3	50.4	74.8	73.3	85.9
Yes	262	60.8	44.5	44.2	74	43.4	75.7	73.8	86.2
p value		0.022	0.549	0.817	0.875	0.163	0.840	0.924	0.940
HbA1c (%)									
<7	377	56.8	37.9	42.7	69.5	41.4	69.8	67.4	83.0
>7	120	85.8	61.7	50.0	87.5	57.2	93.3	93.3	95.8
p value		<0.001	<0.001	<0.001	<0.001	0.002	<0.001	<0.001	<0.001
Blood pressure									
Normal	89	51	32.2	37.3	67.8	35.3	67.8	65.1	80.5
High	205	82.0	60	54.6	82.4	59.5	86.3	85.9	94.1
p value		<0.001	<0.001	<0.001	<0.001	<0.001	<0.001	<0.001	<0.001

^a^ p < 0.001, ^b^ p < 0.010 ^c^ p < 0.05 (two tailed) compared to other subgroups within a category using ANOVA; Dyslipidemia was diagnosed using NCEP ATP III guideline- hypercholesterolemia: total cholesterol (TC) ≥200 mg/dl; hypertriglyceridemia: triglycerides ≥150 mg/dl; low HDL cholesterol: HDL-C < 40 mg/dl (both males and females); high LDL cholesterol: LDL-C ≥ 100 mg/dl; high total cholesterol: HDL-C ratio ≥5; mixed dyslipidemia: when one or more of these lipid parameters were above the cut off values mentioned above

The results of bivariate correlation analysis are presented in Table 4. All correlations were significant at the level of p < 0.001. Both fasting plasma glucose and HbA1c showed significant (p < 0.001) positive correlations with TG, TC, LDL-C, TC/HDL-C ratio, non-HDL-C, ApoB and mixed dyslipidemia except for HDL-C in which case the correlation was negative. TC, LDL, TC/HDL-C ratio and non-HDL-C showed strong positive correlation with ApoB. TC, HDL-C and LDL-C also showed strong correlation with TC/HDL-C ratio. Non-HDL-C and ApoB showed the highest correlation with mixed dyslipidemia.

Multivariate logistic regression models were used to identify the independent predictors of dyslipidemia in the diabetic patients (Table 5). High LDL-C, high non-HDL-C, high ApoB and mixed dyslipidemia were associated with male gender. Only low HDL-C and high TC/HDL-C ratio were associated with central obesity. Likewise, only hypertriglyceridemia and high TC/HDL-C was associated with current smoking habit. Hypercholesterolemia, low HDL-C, high LDL-C, high TC/HDL-C, high non-HDL-C and high ApoB were associated with fasting hyperglycemia. Hypertriglyceridemia, hypercholesterolemia, high LDL-C, high TC/HDL-C, high non-HDL-C, high ApoB and mixed dyslipidemia were associated with poor glycemic control. Likewise, all forms of dyslipidemia except high LDL-C and mixed dyslipidemia were associated with hypertension. Only hypercholesterolemia was found to be associated with duration of hypertension. All associations between dyslipidemia and risk factors were positive except for duration of diabetes in which case the association was negative.

**Table 4:** Correlation among FPG, HbA1c, lipid parameters and mixed dyslipidemia in diabetic patients

Variables	1	2	3	4	5	6	7	8	9	10
1. FPG	–	0.574^**^	0.279^**^	0.258^**^	−0.274^**^	0.223^**^	0.264^**^	0.274^**^	0.270^**^	0.150^**^
2. HbA1c		–	0.376^**^	0.357^**^	−0.157^**^	0.271^**^	0.275^**^	0.344^**^	0.347^**^	0.246^**^
3. TG			–	0.446^**^	−0.309^**^	0.205^**^	0.392^**^	0.452^**^	0.440^**^	0.362^**^
4. TC				–	−0.481^**^	0.954^**^	0.857^**^	0.986^**^	0.990^**^	0.468^**^
5. HDL-C					–	−0.574^**^	**−**0.801^**^	**−**0.595^**^	**−**0.586^**^	**−**0.350^**^
6. LDL-C						–	0.888^**^	0.960^**^	0.966^**^	0.424^**^
7. TC/HDL-C							–	0.913^**^	0.903^**^	0.370^**^
8. Non-HDL-C								–	0.994^**^	0.480^**^
9. ApoB									–	0.489^**^
10. Mixed dyslipidemia										–

^**^p value (two tailed) significant at the level of <0.001. The Pearson’s correlation coefficient (*r*) values of ±1 was interpreted as perfect correlation, *r* values between ±0.7 and ±0.9 as strong correlations, *r* values in the range ±0.4 to ±0.6 as moderate correlations, *r* values between ±0.1 and ±0.3 as weak correlations, and *r* value of 0 as no correlation

**Table 5:** Association of co-variate risk factors independently associated with dyslipidemia in diabetic patients

Risk factors	Hypertriglyceridemia		Hypercholesterolemia		Low HDL-C		High LDL-C	
	OR (95% CI)	p value	OR (95% CI)	p value	(OR) (95% CI)	p value	OR (95% CI)	p value
Age (>55 year)	064 (0.40–1.03)	0.067	0.98 (0.63–1.54)	0.941	1.13 (0.72–1.76)	0.596	1.27 (0.77–2.09)	0.357
Male gender	1.09 (0.69–1.72)	0.720	1.29 (0.83–1.99)	0.245	1.24 (0.81–1.90)	0.324	1.65 (1.03–2.65)	0.039
BMI (>23 kg/m^2^)	1.00 (0.64–1.60)	0.978	1.05 (0.67–1.64)	0.829	0.75 (0.48–1.17)	0.204	1.04 (0.64–1.67)	0.888
Central obesity	1.10 (0.57–2.10)	0.781	1.14 (0.63–2.06)	0.660	4.03 (2.09–7.77)	<0.001	0.83 (0.41–1.70)	0.616
Urban resident	1.08 (0.67–1.73)	0.764	0.92 (0.59–1.44)	0.721	1.14 (0.73–1.78)	0.561	0.83 (0.50–1.36)	0.456
Current smoking	1.77 (1.03–3.02)	0.037	1.07 (0.65–1.74)	0.802	1.15 (0.71–1.87)	0.566	1.07 (0.62–1.85)	0.801
Non-vegetarian diet	1.10 (0.50–2.44)	0.815	0.89 (0.41–1.93)	0.774	0.86 (0.41–1.84)	0.705	1.25 (0.56–2.74)	0.585
Fasting hyperglycemia	1.27 (0.82–1.97)	0.282	2.44 (1.57–3.81)	0.000	3.00 (1.92–4.67)	<0.001	2.16 (1.39–3.37)	0.001
Poor glycemic control	3.86 (2.11–7.07)	<0.001	1.74 (1.09–2.77)	0.020	0.96 (0.60–1.54)	0.874	2.10 (1.12–3.91)	0.020
DM duration(>10 year)	0.65 (0.34–1.23)	0.186	1.08 (0.59–1.97)	0.813	1.12 (0.63–1.55)	0.695	0.86 (0.44–1.69)	0.663
Hypertension (HTN)	4.14 (2.40–7.16)	<0.001	1.97 (1.25–3.12)	0.004	1.93 (1.21–3.07)	0.006	1.46 (0.85–2.51)	0.175
Duration of HTN	1.04 (0.96–1.12)	0.383	1.09 (1.01–1.16)	0.020	1.01 (0.95–1.08)	0.690	1.05 (0.96–1.15)	0.272

Risk factors	High TC/HDL-C		High non-HDL-C		High ApoB		Mixed dyslipidemia	
	OR (95% CI)	p value	OR (95% CI)	p value	OR (95% CI)	p value	OR (95% CI)	p value
Age (>55 year)	1.29 (0.82–2.03)	0.266	0.90 (0.53–1.54)	0.708	0.91 (0.54-1.54)	0.729	0.93 (0.47–1.86)	0.846
Male gender	1.21 (0.79–1.87)	0.383	1.70 (1.03–2.82)	0.038	1.78 (1.08-2.93)	0.023	2.18 (1.13–4.20)	0.021
BMI (>23 kg/m^2^)	0.86 (0.55–1.34)	0.500	0.85 (0.51–1.41)	0.527	0.88 (0.54-1.45)	0.626	1.14 ( 0.61–2.15)	0.679
Central obesity	2.51 (1.35–4.69)	0.004	0.71 (0.31–1.62)	0.422	0.88 (0.41-1.91)	0.750	1.48 (0.54–4.04)	0.448
Urban resident	1.13 (0.72–1.77)	0.602	0.85 (0.50–1.44)	0.542	0.85 (0.50-1.43)	0.530	0.85 (0.42–1.73)	0.660
Current smoking	1.74 (1.06–2.85)	0.029	1.00 (0.56–1.77)	0.989	1.00 (0.56-1.75)	0.985	0.80 (0.37–1.70)	0.562
Non-vegetarian diet	1.12 (0.52–2.41)	0.780	1.20 (0.53–2.76)	0.660	1.21 (0.53-2.77)	0.647	0.96 (0.34–2.74)	0.941
Fasting hyperglycemia	2.92 (1.86–4.57)	<0.001	2.46 (1.55–3.89)	<0.001	2.40 (1.52-3.78)	<0.001	1.76 (0.97–3.16)	0.061
Poor glycemic control	1.33 (0.83-2.15)	0.236	3.92 (1.77–8.63)	0.001	4.60 (2.08-10.01)	<0.001	5.22 (1.52–17.88)	0.009
DM duration (>10 year)	1.04 (0.57–1.90)	0.888	0.58 (0.29–1.15)	0.119	0.51 (0.26-1.02)	0.055	0.40 (0.17–0.91)	0.030
Hypertension (HTN)	2.03 (1.28–3.24)	0.003	1.85 (1.02–3.37)	0.042	1.99 (1.11-3.60)	0.022	2.18 (0.93–5.12)	0.074
Duration of HTN	1.06 (0.99–1.14)	0.081	1.09 (0.98–1.21)	0.095	1.10 (1.0-1.22)	0.056	1.12 (0.95–1.31)	0.162

Values are presented as OR (95% CI). OR = Odds ratio, CI = Confidence Interval. Coding of categorical variables-Age groups: female = 0, male = 1; Sex: female = 0, male = 1; BMI: normal = 0, increased = 1; Central obesity: absent = 0, present = 1; Place of residence: village = 0, urban = 1; Smoking habit: non-smoker = 0, current smoker = 1; Diet: vegetarian = 0, non-vegetarian = 1, Glycemic control: good (HbA1c% <7)=0,poor (HbA1c% >7)=1; Duration of diabetes: <10 years = 0, >10 years, Blood pressure: normotensive = 0, hypertensive = 1. Fasting plasma glucose and duration of diabetes were continuous variables

## Discussion

The aim of our study was to determine the prevalence, pattern and predictors of atherogenic dyslipidemia in a cohort of type 2 diabetic patients from a teaching hospital of Western hilly region of Nepal. We found that the majority of the patients had higher levels of serum TG, TC, non-HDL-C, ApoB and TC/HDL-C ratio and lower level of serum HDL-C than the cut off values recommended by the NCEP ATP III [2]. Abnormal lipid profiles in our diabetic patients were not surprising. Insulin resistance or deficiency leads to increased rate of lipolysis in adipocytes and influx of free fatty acids into the liver resulting into overproduction of triglyceride rich lipoproteins. Moreover there is delayed clearance of such lipoproteins due to decreased activity of the endothelial bound enzyme lipoprotein lipase [19]. There was no significant difference between the serum levels of these lipid parameters between males and females except for serum TG, which is in agreement with previous hospital and population based studies in Asian, African, European and North American type 2 diabetic populations [20–22]. Some studies have also shown higher levels of atherogenic lipid profile in women [22–24] but such different outcomes may have resulted from differences in age distribution, treatment status for diabetes and dyslipidemia, glycemic status, duration of diabetes and nature of study population.

Age is a non-modifiable risk factor for CVD [2]. We next analyzed the effect of age on serum lipid profile of our patients. We observed a rise in the serum levels of TC, LDL-C, non-HDL-C, ApoB and TC/HDL-C ratio with increasing age of patients and a gradual fall in serum TG and HDL-C levels. Several cross-sectional and longitudinal studies conducted elsewhere have also shown similar results [25–27]. The plasma level of lipids is determined by the balance between synthesis and removal of lipoprotein particles. Ageing causes increased TC and LDL-C levels due to impaired clearance from plasma through reduced expression of hepatic LDL-C receptor [28]. Similarly, age-associated rise in ApoB has been shown to be the result of an increased production of VLDL ApoB-100 and decreased clearance rate of LDL-C ApoB-100 [29]. Plasma TG levels were expected to be higher in older patients, but this was not observed in our study. The unexpected decline of plasma TG level with the advancing age could partly be due to masking effect of treatment of certain old age patients with insulin and lipid lowering drugs. Moreover, menopause has been shown to be an additional risk factor in older women that significantly decreases plasma HDL-C and increases LDL-C levels [30]. Age related decline in HDL-C levels likely results from insulin resistance, inflammation, hormonal decline, cellular senescence and ageing of the HDL-C particle itself, affecting HDL-C formation [31]. This explains the increased prevalence of atherogenic dyslipidemia and risk of CVD with age. Our study has confirmed previous findings that serum lipid parameters are highly correlated with fasting blood glucose and HbA1C, irrespective of the population studied. We further observed moderate to strong correlation of primary lipid parameters such as TC, HDL-C, and LDL-C with derived or secondary lipid parameters such as TC/HDL-C ratio, non-HDL-C and ApoB which are regarded as better predictors of insulin resistance, metabolic syndrome and CVDs [32].

There are some studies from other parts of Nepal that have reported varying prevalence and pattern of dyslipidemia in type 2 diabetic patients [20, 33, 34]. The latest prevalence of mixed dyslipidemia was 63.8% in eastern Nepal, 61.0% in central Nepal and 90.7% in mid-western Nepal. The most prevalent single dyslipidemia in both central and mid-western Nepal was low HDL-C. The least prevalent single dyslipidemia was hypercholesterolemia in central Nepal and high LDL-C in mid-western Nepal. Our study provides the first detailed report on the prevalence and pattern of dyslipidemia in diabetic population from the Dhaulagiri, Gandaki and Lumbini zones of western Nepal. We found high prevalence of dyslipidemia in our patients, with mixed dyslipidemia being the predominant type. The most prevalent primary single dyslipidemia was high LDL-C while hypercholesterolemia was the least prevalent. Three quarters of the patient population showed high non-HDL-C. High LDL-C was the only isolated dyslipidemia present in our patients. The typical atherogenic dyslipidemia was present in about half of the patients. Males had significantly higher prevalence of high LDL-C, high non-HDL-C, high ApoB and mixed dyslipidemia than females, while other lipid parameters were similar. This is in agreement with previous reports from Nepal and elsewhere [20, 35, 36]. We found age specific increase in prevalence of dyslipidemia only in males, with females showing stable or decreasing prevalence with age. Our findings are in agreement with previous reports [24]. The age-specific variation in the prevalence of dyslipidemia is believed to be due to age-related decline in sensitivity of peripheral tissues to insulin and increase in metabolic disorders of carbohydrates and lipids [37]. This effect could have been masked in our female patients because of better control of their diabetes.

We also analyzed the effect of other modifiable risk factors of CVD on the prevalence of dyslipidemia. We did not find significant difference in prevalence of dyslipidemia between smoker and non-smoker patients except for hypertriglyceridemia. This lack of difference could be due to inclusion of relatively low number of smokers (27.2%) as compared to non-smokers (72.8%). Another possibility is that the intensity of smoking was low in these smokers and some of the non-smokers may have been recent ex-smokers. Studies have shown that intensity of smoking is associated with small but significant increases in LDL-C and decreases in HDL-C while smoking cessation is associated with improvement in HDL-C, total HDL and large HDL particles, especially in women [38]. As expected, we found significantly higher prevalence of hypertriglyceridemia, low HDL-C, high LDL-C, high TG/HDL-C ratio and mixed dyslipidemia in patients with poor glycemic control and hypertension. Moreover, prevalence of low HDL-C and high TC/HDL-C ratio was also higher in patients with longer duration of diabetes. These findings are in agreement with the findings of many other studies conducted among diabetic patients in other populations [35, 39]. Type 2 diabetes is often associated with the cluster of several other risk factors of CVD such as older age, insulin resistance, obesity, hypertension, poor glycemic status, microalbuminuria, alterations in inflammatory, coagulation and thrombolytic markers in addition to the atherogenic dyslipidemia [40, 41]. The clustering of many of these risk factors often manifests as metabolic syndrome that precedes and then continues with the diabetes [42]. We have previously shown that Nepalese type 2 diabetic patients have high prevalence of metabolic syndrome and increased risk of CHD [43]. We also explored the covariate risk factors that were independently associated with dyslipidemia in our patients. Age group ≥55 year, current smoking habit, fasting hyperglycemia, poor glycemic control and hypertension were found to be strongly associated with hypertriglyceridemia. Fasting plasma glucose, hypertension and its duration were associated with hypercholesterolemia. Low HDL-C was associated with male gender, central obesity and fasting plasma glucose. High LDL-C was positively associated with male gender and fasting plasma glucose. High non-HDL-C and ApoB were independently associated with male gender, fasting plasma glucose, poor glycemic control and hypertension. High ApoB was also found to be associated with longer duration of hypertension. These risk factors are recognized by many international guidelines and remain the targets for preventing CVDs among diabetic patients. As dyslipidemia is a well-established risk factor for cardiovascular diseases, presence of other co-variate risk factors results significantly higher risk of future CVD. This is in agreement with our earlier studies which have estimated higher risk of 10-year CHD among Nepalese type diabetic patients [43]. These findings warrant extensive preventive approaches, both clinical and non-clinical, to treat all types of dyslipidemia to minimize the future risk of CVD in our patients. Government and other stakeholders of the health need to collaborate to increase public awareness about the impact of atherogenic dyslipidemia among both patients and health care providers and promote adoption of healthy diet and life-style.

### Study limitations

The major limitation of our study is that it included only hospital based samples which may not truly represent the diabetic population of this region. Second, it did not analyze the types and effect of lipid lowering treatment in the dyslipidemic patients. Third, it also overlooked the analysis of the differences in diabetes treatment in patients with and without dyslipidemia. The findings of this study, therefore, should be interpreted within context, and may not be generalized to the whole diabetic population.

## Conclusions

Our study provides the first detailed report of prevalence, pattern and predictors of atherogenic dyslipidemia in type 2 diabetic patients attending a tertiary care hospital of western hilly region of Nepal. It has shown an alarmingly high prevalence of dyslipidemia. Mixed dyslipidemia is more prevalent than combined or single dyslipidemia. The prevalence of dyslipidemia was found to be strongly associated with various co-variate risk factors of CVD such as old age, male gender, smoking, poor glycemic control, obesity and hypertension suggesting high risk of future CVD. Our study therefore contributes to the epidemiology of diabetic dyslipidemia from the Western hilly region of Nepal and serves as supportive data for health policy planners to formulate and implement policies that aim to increase public awareness about diabetic dyslipidemia, healthy diet and life-style among diabetic patients and health care providers. It also highlights the need of regular monitoring of blood glucose and lipid profile, aggressive lifestyle changes such as weight reduction and physical exercise and effective medication with anti-diabetic and lipid lowering drugs to obtain proper glycemic control and lipid profile. However, a population based nationwide survey is still warranted to reflect the actual epidemiology of diabetic dyslipidemia in Nepal as no such studies has been carried out so far.

## Additional file

**Additional file 1.** Survey questionnaire and data collection form for screening dyslipidemia in Nepalese individuals with type 2 diabetes.

## Abbreviations

ANOVA: analysis of variance
ApoB: apoliprotein B
BMI: body mass index
CHD: coronary heart disease
CVD: cardiovascular disease
EDTA: ethylene diamine tetraacetate
HbA1c: glycated hemoglobin type A1c
HDL-C: high density lipoprotein cholesterol
IRC: institutional review committee
LDL-C: low density lipoprotein cholesterol
MTH: manipal teaching hospital
NCEP ATP III: the national cholesterol education program adult treatment plan III
SD: standard deviation
SPSS: statistical package for the social sciences
T2DM: type 2 diabetes mellitus
TC: total cholesterol
TG: triglycerides
VLDL: very low density lipoprotein
