# Supplementary material for: Prevalence and pattern of dyslipidemia in Nepalese individuals with type 2 diabetes
Source: BMC Res Notes. 2017 Apr 4;10:146. doi: 10.1186/s13104-017-2465-4 (PMC5379598; doi:10.1186/s13104-017-2465-4)
Supplement: Supplementary file 1 — Additional file 1. Survey questionnaire and data collection form for screening dyslipidemia in Nepalese individuals with type 2 diabetes. [file 13104_2017_2465_MOESM1_ESM.docx]

# Survey Questionnaire and Data collection form

**Title of the study:** Prevalence and Pattern of dyslipidemia in Nepalese individuals with type 2 diabetes

# Principal Investigator: Dr. Daya Ram Pokharel

# Department of Biochemistry

# Manipal College of Medical Sciences

# Pokhara-16, Kaski

# Nepal

**Study site:** Manipal Teaching Hospital, Phulbari, Pokhara

**Personal details**

**Patient Name: ____________________ Age**: _______ **Gender: (M / F) Hospital no._________**

**Address:** Ward no.: VDC/Municipality: District:

Tel/Mobile: Email:

**Anthropometry**

**Body weight (kg):** **Height (cm): Waist (cm):**

**BMI (kg/m^2^):** BP (mmHg, average of ≥2 measurement): Systolic---------- Diastolic:----------

**Socio-demographic variables**:

**Religion:** □Hindu □Buddhist □Muslims □Christians □Others (specify):

**Ethnicity**: □Brahman □Chhetri □Vaishya □Dalit □Gurung □Magar □Thakali □ Bhote/Sherpa □Newar □Tharus □Others (specify):

**Geographical origin**: □Madhes □Pahad □Himal

**Marital status**: □ Married □ Unmarried □ Divorced □ Widowed

**Educational qualification:**

□No formal education □Primary (upto class 8) □Secondary (class 9-12) □University

**Occupation:**

□ Agriculture □Business □Office job □Security job (Army, police etc) □Housewife

□ Factory worker/Laborer □Pensioner □Others (Specify):

**Dietary habit:** □Vegetarian □Non-vegetarian

**Types of meat most frequently consumed (if non-vegetarian)**

□Red meat (Mutton/Buff/Pork) □Chicken □Fish □No preference/any type

**Alcohol drinking habit:** □Regular drinker □Occasional drinker □Non drinker □Former drinker

**Smoking habit:** □Current smoker □Ex-smoker □Non smoker □Passive smoker

**Duration of smoking (years):**

**Current smoker:** □0-5 □5-10 □10-15 □>15

**Ex-smoker**: □0-5 □5-10 □10-15 □>15

**Passive smoker**: □0-5 □5-10 □10-15 □>15

**Daily physical exercise/activity:** □Regular □Occasional □Never

**Daily average duration of physical activity (hour):**

**Relevant clinical history**

**Do you have diabetes mellitus (DM)?** □Yes □No (Please verify it from the personal medical records or fresh results of the plasma glucose estimations)

**If yes, age at diagnosis (years):**

**Type of DM:** □Type 1 □Type 2 □Others (specify)----------------------- □Not known

**Total Duration of DM (years):** □0-5 □5-10 □10-15 □>15

**Family history DM:** □Yes □No □Do not know

**Treatment status of DM**: □No medicine, self control □Metformin □Sulfonylureas

□α-glucosidase inhibitors □Thiazolidenidiones □Insulin

**History of hypertension (HTN):** □Yes □No (Please verify it from the personal medical records or BP measured during this interview)

**Age at diagnosis (years):**

**Duration of HTN (years):** □0-5 □5-10 □10-15 □>15

**Treatment status of HTN: □**Yes □No

**Presence of dyslipidemia:** □Yes □No (Please verify from the personal medical records or fresh fasting lipid profile results)

**Treatment for dyslipidemia:** □Yes □No

**History of current or previous CVD events:** □Yes □No

**Thank you for your participation in our research study!**

**Name of Data Collector: Signature of Data Collector:**

**Date:**
